# Defining complementary and alternative medicine: Revisiting the Debate and Plea for a Strategy Based on Plausible Effectiveness

**DOI:** 10.1007/s11019-025-10291-6

**Published:** 2025-08-13

**Authors:** Alexander Kremling, Jan Schildmann

**Affiliations:** Interdisciplinary Center for Health Sciences, Institute for History and Ethics of Medicine, Martin Luther University Halle-Wittenberg, Halle (Saale), Germany

**Keywords:** Complementary and alternative medicine, Explication, Terminology, Definition

## Abstract

Discourse about Complementary and alternative medicine (CAM) is also controversial in several respects, including terminology. Understanding and using the term ‘CAM’ precisely remains necessary in some discussions. This article provides a contribution to a terminologically more reflected debate about CAM. Analytical methods are applied to analyse ‘CAM’ conceptually: reasons to define CAM are discussed, common definitions are critically analysed in light of argumentative plausibility, and typical conceptual needs in the debate about CAM are described. Based on this, an evidence definition of CAM is sketched. Complementary and alternative medicine is typically defined by positive attributes or (more usefully) by unconventionality. While the latter provides a viable definitional strategy, several questions remain regarding the logic and applicability. Attempts to improve CAM definitions should consider (a) presenting necessary and sufficient conditions, (b) separating ‘complementary’, ‘alternative’ and ‘integrative’, (c) understanding ‘CAM’ relative to specific diseases and (d) being explicit about possible changes of the CAM status. These requirements are used to develop a definition of CAM centring around the notion of probable specific effectiveness—a definitional strategy that might solve flaws in the current CAM discourse by spelling out some of the reasons why certain practices are not part of conventional treatment. The example of the cancer drug Imatinib serves to demonstrate the usefulness of focusing on plausibility of effectiveness instead of conventionality. Defining CAM in light of evidence properties might improve the debate. Independent of the terminological strategy pursued, articles and guidelines on CAM should at least reflect the implications and pros and cons of their own terminological decision. An evidence definition should be developed in detail.

## Why is a good complementary and alternative medicine (CAM) definition important?

1 Historically, “alternative medicine” was the term used more prominently from the 1990 s on to, to establish a category for treatments that had until the been labelled by clearly pejorative terms like “non-scientific” or “charlatanism”. The terms “complementary” and “integrative” followed (Ng et al. 2023). The term “CAM” dominates discourse since year 2000, compared to other expressions like “complementary” or “alternative medicine” alone or “integrative medicine” (Ng et al. 2016). Treatments^2^ that are typically labelled as ‘complementary’ or ‘alternative’ – CAM – are very important for many people. Debates about theses treatments touch overlapping topics of autonomous decisions about taking care of one’s health, trust in official medical recommendations and the role of science in the intimate sphere of personal health. It is no wonder that CAM discussions have a tendency to move people’s minds in both everyday life and academia (Kremling et al. 2024).

At first glance, defining CAM appears to be a logical necessity: no scientific discourse about anything without clarifying what the discourse is about. However, it also seems viable to only talk about groups of treatments with shared properties even without sticking any particular label on them. ‘Treatments with a low risk of severe direct adverse effects but inconclusive evidence about specific efficacy’ or ‘treatments that are requested by patients but have an effect only as good as placebo interventions’^3^ would be examples. Some treatments in these groups might often be labelled as CAM – but why not discuss them independently and only in light of the properties in question? It seems possible to break down discussions about CAM into well described subsets of treatments without bothering about a terminological label and its boundaries.

Some discourses would profit from this reduced (‘deflationary’) and phenomena oriented strategy, because unnecessary and potentially ideological discussions might be avoided. Others nevertheless would not. Those are discourses requiring the ‘classificatory’ (structuring, organizing) function of a definition. Can a clinic’s budget for complementary medicine be spent on physiotherapy? Which interventions should be selected for evaluation in a systematic guideline, for example on complementary medicine in oncology? Is the new professor for complementary and alternative therapies entitled to teach both homeopathy and nutrition counselling, or only one of them? Those are considerations of drawing lines (also) based on terminology.

The assumption is that there are legitimate speech interest about CAM “in general” that legitimize focussing on better terminology or at least make an attempt prima facie worth trying.^4^ Studies like the one of Kemppainen and Kemppainen (2025) about the development of CAM use in Europe or about communication about CAM with cancer patients (Wode et al. (2023)) serve a general interest that goes beyond separate interests about the “individual” mentioned treatments.

Being considered in- or outside of complementary medicine can have significant practical consequences for a treatment in practical contexts. A change of terminology might also effect public discourse – though public discourse and use of terms is different from more regulated academic discourse and probably also more resistant to change. Relevant (at least implicit) questions for patients might be: Who is reliable in case of my health issue? Who can be trusted on which grounds? How can I identify the line between best available knowledge and probably unnecessary time and effort as well as potential harm.

We thus assume that it is necessary to use the expression ‘CAM’ at least in some discussions, and have an understanding of how to use the term as clearly as possible.

### Refraining from defining CAM is not a definitional strategy

As described above, it is worth considering not defining at all and accepting that the concept is ‘fuzzy’ and vague in opposition to conventional medicine. Schöne-Seifert et al. (2015) adopt this position and discuss the pros and cons. The basic idea is to accept that it will not be possible to come to a pacifying definition that satisfies everybody involved in the discourse, which could be particularly important in a context of diverse backgrounds and robust dissent in CAM discourse.

This strategy implies that even contradictory implicit definitional criteria are accepted and (in a way) embraced – instead of a “profound attempt to regulate speech” (Schöne-Seifert et al. 2015, 238, translated by the authors).^5^ This is not a definitional strategy, but rather refraining from defining CAM at all. This strategy results in a neutral ‘anything goes’ situation that mirrors, in a way, the factual use of the concept for numerous purposes from various and inconsistent backgrounds. Schöne-Seifert et al. accompany this strategy by warning about a class of ‘irrational-doctrinaire CAM-treatments” – a class they probably define implicitly as treatments that are offered despite sufficient evidence of ineffectiveness (Schöne-Seifert et al. 2015). They present this as an “internal differentiation” in the CAM class. This can be understood as a description of a precise subclass of the fuzzy CAM class. However, this can also be understood as an attempt to at least describe a sufficiently precise set of treatments (“irrational-doctrinaire treatments”) covering those treatments that Schöne-Seifert et al. deem to be highly important and that merely has an overlap with the fuzzy CAM class. Classificatory questions though cannot be answered in this way. Being ‘CAM’ does not imply anything if it is explicitly undefined.

One motivation or related perspective to not defining CAM would be the attempt to get rid of the concept at all. This position goes beyond the thoughts of Schöne-Seifert et al. but is nevertheless worth considering. We think though that given the far-spread use of “CAM” as a term in academic and public discourse as well as the fact that there are several indications for legitimate general interests about this topic, exploring chances to improve the concept is the better (or at least a prima facie legitimate) strategy.

## Strategies of defining CAM and critique

Defining CAM at all, thus, can be questioned, but in those publications where CAM actually is defined, the definitions differ in detail. Nevertheless, two common strategies to define CAM can be distinguished, of which only one is a viable definitional option, while the other one can be criticized.

We would like to comment on lists of CAM-therapies before discussing these two options – in particular whether is also a viable strategy to “just” list therapies as an approach to clarify the concept. The approach would be to state “Here, CAM is $\{t_1, t_2, t_3,...t_n\}$”, with *t* being listed therapies. However, applying a definition will always result in sets of items: Items that fulfil the definition and items that do not fulfil it (and maybe, if one accepts such a class, a set of items for which the status is unclear). Thus, a definition of a class of therapies and a list of therapies are not two entirely unrelated things. If only a list is provided the obvious question is: How was this list generated? If a list is provided without the rules that produced them, then the list depends on other author’s or institution’s definition of CAM. If the list was produced by applying a set of rules, then these rules or conditions are the definition “in action”.

This is of special importance when authors present a list of therapies but have indeed a set of criteria with which they are producing the list. In such a case the list is best understood as the “result” of the author’s definition and not itself an “enumerative definition”. The account of Wieland et al. below will be a good example of this. Mere enumeration also leaves the therapies conceptually unrelated and provides no tool to smoothly include or exclude items based on criteria. Understanding (and maybe also critically challenging) the connection of the items on the list is not possible.

### Defining by listing positive attributes?

Providers of CAM sometimes characterize CAM with positive attributes. Those characterizations might be misunderstood when understood as definitions in a logical sense. In the case of the website of the European Federation for Complementary and Alternative Medicine though, context and headline (“CAM definition”) as well as absence of anything else but the following list of attributes, suggest that the list is indeed meant as a terminological clarification of what CAM is:

Definition of CAM

A diverse range of autonomous healthcare practices used for health maintenance, health promotion, disease prevention and for the treatment of ill-health. These practices can be used independently, and, alongside conventional medical approaches to create a broad range of healthcare options for the public.

CAM’s particular strength is the combination of individualized holistic care, capacity to provide health maintenance, illness prevention and non-invasive illness treatment as part of an integrated package. This is very attractive to users who report a high satisfaction rating.

CAM practices are:

- Holistic
- Natural
- Curative
- Preventive
- Health supporting
- Open to innovation
- Traditional and established
- Safe for both patients and users
- Enhancing of self-healing capacities
- Promoting of self-responsibility for health
- Able to be used either in combinations or individually
- Increasing the range of options available for patient care and treatment

European (2023)

The properties listed do not result in a distinctive definition, neither by conjunction (...and...) nor adjunction (...or...). The list, instead, represents positive aspects and effects that a yet to be disclosed set of interventions – EFCAM only cites acupuncture, shiatsu, traditional Chinese medicine and homeopathy as examples – is supposed to have. Let alone that each entry is not a plausible distinctive feature, a list like this will not fulfil the classificatory function of a definition. The practical correlate of this missing clear definition is: Which treatments is EFCAM responsible for? Which treatments does someone support who donates to EFCAM? A list of positive attributes is not a helpful definitional strategy to answer these questions.

### Defining by unconventionality

The most common strategy to define CAM is to elaborate the idea that CAM is ‘unconventional’. This is in line with the definition used by the World Health Organization in their publications – though it has lately been removed from their websites:

Complementary medicine (CM):

The terms “complementary medicine” or “alternative medicine” refer to a broad set of health care practices that are not part of that country’s own tradition or conventional medicine and are not fully integrated into the dominant health-care system. They are used interchangeably with traditional medicine in some countries. (World Health Organization 2013)

Others have used their own words for the same idea (Carroll and Richard 2007, 10), while not providing more thoughts about the crucial expressions or or have even declared that precision is impossible (Chatfield and Kate 2018, 1). Wieland et al. (2011) though formulated an “operational definition” for the Cochrane Collaboration in line with this definition in order to generate a list of CAM interventions for databases with keywords.^6^ The list was updated and reorganized in a recent publication (Ng et al. 2022), which, nevertheless, relies on the same definition.^7^ The costs and consequences of this definition (in the sense of potential negative impact on CAM discourse and logical implications) have, to the best of our knowledge, not yet been discussed, though it is often cited in the CAM discourse.^8^

A first observation is that the definition is not clear, in the sense that it can be interpreted in different ways. A plausible reading is that it consists of three conditions: $a =$ ‘developed outside of the Western medical model’ (“allopathic medical model”, (Wieland et al. 2011, 5))^9^, $b=$ ‘currently not considered standard treatment’, or $c=$ not “delivered exclusively by conventionally credentialed medical personnel or exclusively within hospital settings”. Conditions for something to be considered standard treatment are “government licensing of practitioners, coverage by health insurance, statements of approval by government agencies, and recommendation as part of a practice guideline” (Wieland et al. 2011, 5). All three conditions are negative ones. CAM is something that does ‘not’ have certain properties.

The authors remain vague about whether they take their third condition (*c*) to be a sufficient one (i.e. one that definitely implies a CAM status), but if so, then their suggestion can be interpreted as a jointly necessary adjunction “*a* or *b* or *c*”, so that fulfilling at least one condition is sufficient for a treatment to be CAM, and being CAM implies that at least one condition is fulfilled.

A consequence of (a) is a strict historical rule. The definition implies that no matter how established a practice might be today – if it was developed, for example, by an indigenous culture before Western influence or colonization (take the example of using opium poppy), it is “unconventional” according to the authors, consequently CAM and inevitably CAM forever. A change of status is impossible given their first condition (and the adjunctive interpretation). This comes together with an inherent historical vagueness: What is the “Western medical model”? We doubt that it is possible to give precision to this that lives up to the history of medicine.

In light of this historical perspective, it is also worth noting that the authors made the odd decision to use the expression “allopathic” to clarify the expression “the conventional Western medical model [of disease and healing]” (Wieland et al. 2011, 4).^10^ ‘Allopathic’ is a combat term of Samuel Hahnemann, the founder of homeopathy, meaning ‘everything that is not homeopathic’ in his time. The authors probably did not want to subscribe to a 19th century homeopathic view of medical history. The term does not clarify much. Are heroic therapies, following principles of humoral pathology (which is what Hahnemann had in mind) part of ‘the Western medical model’?

Fulfilment of (b) and (c) might change over time but the perspective of this matter is non-normative, observing factual institutional practice and integration. Regarding (b) and (c), we wonder how partial integration into a healthcare system is evaluated in detail, how the several indicators are offset against each other, and how novel treatments are evaluated. The remaining vagueness of the indicators for “standard treatment” might leave a lot of space for interpretation. We also do not fully understand the ‘not exclusively within hospitals’ condition, because this appears to us to be a counter-intuitive condition for ‘non-standard’: Why should treatments provided by general practitioners be, per se, considered CAM?

To sum up, several questions remain about the logic and applicability of the definition. From a distant perspective it also seems only natural to dismiss the strategy to only observe and describe factual acceptance and the resulting problems and instead, spell out the good reasons for acceptance of a treatment as definitional criteria. These reasons might be – at least in a relevant amount of cases – the causes for treatments being integrated into or disintegrated from standard healthcare. A perspective on (good) reasons for conventionality is, in this way, a logical next step after focussing on factual conventionality. This would result in an evidence definition of CAM, i.e. a definition that uses evidence, not conventionality, as a key definitional criterion. Wieland et al. (2011) dismiss this strategy in their article – perhaps too hastily, as we will argue below.

## Preliminary criteria for a good contribution concerning CAM terminology

Before attempting an evidence definition of CAM, we want to discuss general ‘points to consider’ for any definition following whatever strategy that increase the chances to make a valuable contribution to the debate. Each point is arguable and requires its own, sometimes maybe extensive, defence. However they are in this article, due to a lack of space, assumed to be plausible. There may also be other points but these give at least a first guide to discuss and evaluate terminological CAM suggestions:

1. Presenting the definition as necessary and sufficient conditions
2. Separation of ‘complementary’, ‘alternative’ and ‘traditional’
3. Understanding ‘CAM’ as an umbrella term for indication specific treatments
4. Explicitness about the possibility of changes of the terminological status of a treatment

### Necessity and sufficiency

The understanding of ‘definition’ and ‘condition’ here refers to standards in general philosophy of language, especially logic and argumentation theory, independent from technical details about the exact set of rules that definitions have to fulfil. Definitions there are biconditionals, expressed not by an ‘... is...’-sentence or ‘if... then...’, but by ‘if... and only if... then...’ or a respective symbol or expression (‘iff’, ${:=}$). Regarding the CAM debate, this means that if a treatment fulfils the conditions mentioned in the CAM definition, it is CAM – fulfilling the criteria is logically *sufficient* to qualify as CAM. The other way around it means that if a treatment is CAM then it fulfils the conditions mentioned in the CAM definition – it is logically *necessary* for CAM treatments to fulfil the criteria. This is settled by the definition as a rule. A CAM definition in this sense is a rule about when to use the expression “*X* is CAM”. Such a rule does not have to match everybody’s way of talking about CAM. Instead, it is enough to have a partial overlap of typical ways to talk about CAM and the talk about CAM implied by the definition, if the definition is useful. There are established theories of definitions and also concept clarification (explication) in the philosophy of language and general theory of science (Cordes et al. 2018) to which we refer but which we can not defend or discuss here. For the purposes of this paper, we will assume that this stricter, more regulated definition and its implications are helpful tools for bringing clarity and inferential commitment to the debate.

The definition given by [Falkenberg et al. (2012)7] fails in this respect: The description of CAM they provide only contains unspecific properties that are neither necessary nor sufficient. It is, for example, explicitly mentioned that CAM therapies can have a unconventional or conventional status:

Complementary and Alternative Medicine (CAM) utilised by European citizens represents a variety of different medical systems and therapies based on the knowledge, skills and practices derived from theories, philosophies and experiences used to maintain and improve health, as well as to prevent, diagnose, relieve or treat physical and mental illnesses. CAM has been mainly used outside conventional health care, but in some countries certain treatments are being adopted or adapted by conventional health care.

### Separating complementary, alternative and traditional

Authors may define ‘complementary’, ‘alternative’ and ‘traditional’ (and perhaps even ‘integrative’) all together. Ng et al. (2022), for example use the acronym ‘CAIM’ (complementary, alternative and integrative medicine) instead of ‘CAM’, thereby adding ‘integrative’ to CAM, sparing ‘traditional’. The possible negative consequences of combining several expressions in one technical term (here: CAIM) are so strong for a structured discourse that good reasons should be given to do this. The intuitive differences between the three terms in ordinary language are so large that only providing one definition for all will cause confusion. ‘Complementary’ will be intuitively linked to ‘alongside’ or ‘together with’, ‘alternative’ will be intuitively linked to ‘instead of’ or ‘as another option’ and ‘traditional’ will be intuitively linked to bygone ages. This should be reflected in terminological clarifications.

### ‘CAM’ as an umbrella term for indication-specific treatments

Some patients use vitamin supplements, for example vitamin C in an attempt to treat their cancer or prevent a relapse. From a scientific standpoint, this is (depending on the definition) an alternative or complementary treatment strategy. Nevertheless, vitamin C is the best treatment option to prevent or cure scurvy for people who are malnourished. There may be a confusion now about whether vitamin C is CAM or not. In one respect it is, in another it is not. A simple solution is to describe vitamin C for treating cancer as CAM, but not as CAM for treating scurvy. ‘CAM’ would be the umbrella term subsuming all treatments with CAM status relative to a certain condition. Authors of a terminological proposal should clarify how they suggest handling such terminological situations, which seem to occur regularly (see “calcium”, “cognitive therapy”, “laser therapy”, “plant-based medicines”, “radiation therapy“, “vitamin A” or “water aerobics” in Ng et al. (2022)). The cases can also be more difficult than vitamin C concerning evidence (e.g. acupuncture).

### Changes in terminological status

A terminological contribution should clarify whether a treatment (for a condition) has its ‘CAM status’ once and for all, or whether this can change. If the status can change then the reasons for such a change should be clearly connected to the conditions mentioned in the definition. It should also be transparent whether treatments (for a condition) can have a status ‘in between’ or an unclear status depending on some conditions mentioned in the definition.

## Evidence and beyond: a suggestion for an improved CAM definition

### Defining CAM by evidence (“evidence strategy”)

Defining CAM by a deficiency in the evidence available is rarely done explicitly. It can be ascribed nevertheless to those authors who discuss CAM as ‘unscientific’ or ‘not science based’, since ‘scientific’ implicitly often stands for fulfilled standardized evidence (or plausibility) criteria.^11^

There are arguments, however, against defining CAM by evidence. Wieland et al. (2011), for example, reject this strategy before putting forth their definition based on conventionality:

[W]e should mention that we did not consider evidence of efficacy (or lack of evidence) as a test for identifying a CAM therapy. This is because there are many therapies that are not currently accepted as efficacious, but not all of them would be necessarily considered CAM. For example, a new synthetic chemotherapy agent would not be considered CAM, even if it has not been proven to be efficacious, while an herbal therapy for cancer would generally be considered CAM, even where it had trial evidence of efficacy. (Wieland et al. 2011, 6)

The argument seems to be that a large set of treatments are intuitively and typically called CAM that would not be called CAM according to an ‘evidence strategy’ that the authors anticipate here. In addition, a large set of therapies would have to be called CAM although they are intuitively and typically not considered CAM. The authors claim unwanted consequences of the evidence strategy that would undermine the acceptability of any such definition. They claim that this might involve about 75% of all treatments, because this percentage does not satisfy Cochrane’s effectiveness criteria. From the perspective of a theory of explication, this is the claim that the set of treatments commonly labelled CAM and the set of treatments satisfying an evidence definition would differ so significantly that adopting the definition would distort discourse. The explication would not pass the test for adequacy (Cordes et al. 2018).

Is this argument sound? An actual definition following the evidence strategy instead of a merely anticipated one would be of help to assess this, mainly because the argument anticipates evidence strictly as highest level evidence following evidence-based medicine standards. Maybe an explicit evidence definition could avoid obvious counterarguments. The (sketched) argument from Wieland et al. (2011) seems to depend on the standards for effectiveness applied and whether it is possible to spell out a scientific concept of ‘plausibility of effectiveness’.

There should be more to say about effectiveness than just adopting the position: Passing an evaluation according to the gold standard of highest quality meta-studies of randomized controlled trials implies effectiveness, and not satisfying this standard implies the same status as any randomly generated fantasy treatment. It is possible to take a closer look at the sum of available knowledge about a treatment and discuss the answer to a question such as: What is the initial body of evidence of some new drug therapy against cancer?

### Assessing plausibility of effectiveness: the example of Imatinib

We want to answer this question by looking at the example of Imatinib against chronic myelogenous leukaemia, one of the first so called ‘targeted treatments’, assuming a situation before the clinical tests on humans proving its efficacy (O’Brien et al. 2003, the IRIS-Study). The evidence base in this situation was different than, let us take an esoteric example, the evidence of quantum healing for the treatment of cancer, though we did not have any peer-reviewed systematic reviews of randomized controlled trials in either cases.

In the case of Imatinib (in the assumed situation), there are several hard facts qualifying Imatinib as a highly plausible candidate for curing, without guaranteeing effectiveness in the end. Most importantly:

- Imatinib has effects on the cellular level that match the independently established cause of chronic myelogenous leukaemia
  - Chronic myelogenous leukaemia can be identified physiologically as an high increase and proliferation of leukocytes
  - The cause of this is the formation and then expression of a specific fusion gene (BCR-ABL1 on the so called ‘Philadelphia chromosome’) for most of the patients
  - Imatinib interferes with the biochemical reactions of the resulting pathogenic enzymes causing the death of those cells
  - No specific biochemical mechanism was known to probably counteract this mechanism in the human body

The list roughly describes bits of knowledge as a set of biochemical causal relationships that were based on reliable studies before a phase II study testing effectiveness. The probability of the effectiveness was high because of a tight position of effect-related findings in the scientific “web of beliefs’. Speaking from a position of theory of science and especially theory of argumentation, the claim that Imatinib is effective has a higher connectedness with true statements about cancer and cancer treatment and therefore a higher ‘net probability’ of being true compared to the claim that cancer can be healed by prayer, homeopathic globules or reflexology massages.^12^

Today, the effectiveness of Imatinib and other targeted cancer treatments is itself a strong ‘knot’ in the scientific web of beliefs. We cannot discuss here that this can also lead to an overestimation of the plausibility of effectiveness of novel substances. We want to emphasize that the proven effects of Imatinib and similar substances do indeed increase the initial probability of effectiveness of similar substances initially – which is, nevertheless, different from a proof of effectiveness.

We intend to demonstrate with this example that a new synthetic chemotherapy could pass an evidence criterion for not being CAM – despite not satisfying ‘systematic review’-criterion assumed by Wieland et al. (2011). Such therapy attempts could be called ‘experimental treatments’, candidates for standard treatment with yet insufficient data but a sufficient initial amount of curative plausibility – independent of whether the plausible effectiveness turns out to be actual effectiveness after undergoing a proper research process. To put it straight as a challenge to the argument in the quote in Wieland et al. (2011): The case of Imatinib shows that it did not have to be called CAM as a consequence of an evidence definition of CAM just because it had not yet been tested in clinical trials.

Concerning the other sketched example in the quote – an effective herbal therapy of cancer that some people call complementary or alternative: Perhaps it is a good idea – a price worth paying – to ‘not’ call a sufficiently tested therapy CAM. Under ideal circumstances, we would base all conventional integration of treatments (including interventions tailored to subgroups of patients) on proper studies. Unfortunately, the scientific situation is not ideal, with examples of unnecessary, useless or even harmful interventions. From the perspective of the evidence strategy, the consequence would be that some highly accepted treatments are, in fact, CAM, while others might be considered CAM now but might lose this label in the future in light of new, positive evidence.

The intuition behind the evidence strategy is simple: There are probably, at least for a majority of treatments, reasons (not only causes) why certain practices are not part of standard treatment. Given the self-proclaimed focus on methods of evidence based medicine, these should ideally be reasons concerning evidence about effectiveness. The argument given against it seems weak enough to give the evidence strategy a try.

Another important argument against an evidence strategy is given by Schöne-Seifert et al. (2015). The idea is, that an evidence definition would disrupt or escalate discourse because its main thrust would not be accepted by participants in the discourse that ‘like’ certain CAM therapies. The authors think that those participants would see CAM delegitimized by definition. This argument draws attention to the fact that definition of central terms in a debate is an intervention that can affect this discourse negatively. The argumentation, nevertheless, more or less neglects that an evidence definition is also a permanent invitation to review evidence and get rid of the CAM status as soon as the evidence is sufficient. This would nonetheless have to be communicated properly.

Concerning their objection that an evidence definition would be an “attempt to regulate speech” (see above), we would like to assess the pros and cons again and consider that every conceptual explication will result in a regulation of speech, and the logical extensions of the concepts before and after explication will be different. This should not be done without need, but a clearer terminological situation with fewer misunderstanding or agendas hidden behind vague concepts seem to be worth considering changing CAM terminology.

### Development of and discussion of an explicit definition

Each definitional strategy comes with a cost, in the sense that it has particular weaknesses or blind spots and will probably ex- or includes one or the other treatment in a counter-intuitive way. Given the current confusion about CAM, these effects could be lower when following the evidence strategy.^13^ Without precision, i.e. merely for heuristic purposes, CAM can be characterized as medicine with implausible effectiveness that is, however, used instead of (alternative to) or alongside (complementary with) medicine with plausible effectiveness. ‘Plausibility’ is the central vague term in this heuristic characterization. Schöne-Seifert et al. (2015) have suggested a hierarchy of plausibility levels that give an impression of a more precise account of plausibility. They distinguish the following^14^ (1) validated interventions that have a statistically demonstrated or clinically dramatically evident effect with (1a) a sufficient scientific explanation (such as insulin for type 1 diabetes) or (1b) without a sufficient scientific explanation; (2) non-validated interventions with (2a) a highly plausible effect according to established scientific causal knowledge that are so plausible that they are not even being considered for validation or (2b) non-validated interventions, at least plausible enough for testing; (2c) non-validated interventions too implausible to qualify for nevertheless possible testing; (2d) non-validated interventions not testable according to proponents of the intervention; (3) interventions with sufficient statistical evidence for inefficacy that (3a) have some degree of theoretical plausibility or (3b) are also theoretically highly implausible (e.g. homeopathy).

Future research could add criteria for theoretical plausibility in such a way that the impression is avoided that this is a subjective category depending on taste and personal preference or – again – conventionality. Following the theory-independence of evidence-based medicine, we think that theoretical plausibility is an important concept, but only as a – sometimes necessary and reasonable – shortcut to assess the plausibility that an intervention will have an effect. Theoretical plausibility serves as a fallible indicator of efficacy, so that not everything has to be tested – see (2a) and (2b).

We would like to now move from intuitions to a sketch how an explicit CAM definition following this evidence strategy could look like. The following definitions are suggested to provide a sketch of an evidence definition for the explicatum “CAM” (*C*) based on a definition of “complementary or alternative health measure” (*D*), a disambiguation of complementary (*E*) from alternative health measures (*F*) after providing not definitions but at least criteria for the two central concepts used in these definitions: “probably not effective” (*A*) and implausibility (*B*):

*A*: A health measure *x* is **probably not effective** if (a) clinical studies on effectiveness have been conducted but did not show a specific effect or (b) specific effects have not been sufficiently studied but are implausible.
*B*: Specific effects of insufficiently tested health measures are **implausible** if neither the basic mechanism of the measure can be reproduced nor is there an effective analogous intervention nor a successful (*in vivo/in vitro*) experimental model of the intervention.
*C*: **Complementary/Alternative medicine** is the name of the medical discipline concerned with complementary or alternative health measures as defined below.
*D*: *x* is a **complementary or alternative health measure to treat/prevent/diagnose disease/symptom/condition** *a* if and only if (1) *x* is used to treat/prevent/diagnose *a* but (2) *x* is probably not specifically effective in this respect.
*E*: *x* is a **complementary health measure** to treat/prevent/diagnose disease/symptom/condition *a* if and only if the use of *x* for this purpose is, in addition to (1) and (2), justified.
*F*: *x* is an **alternative health measure** to treat/prevent disease/symptom/condition *a* if and only if the use of *x* for this purpose is, in addition to (1) and (2), not justified.

The complementary medicine status would depend on the use of probably not effective interventions being ‘nevertheless justified’ in a treatment, prevention or diagnostic setting. This would include a careful evaluation from the perspective of medical ethics including topics such as legitimate placebo treatment (Bildik 2024, cf).

The alternative medicine status would depend on the use of probably not effective interventions for which there is also no (other) reason for justified use. This adds a normative justification to the intuition that alternative medicine contradicts (reasonable) medical practice. Here this would not be a contingent but a terminological matter. A measure would loose its alternative status as soon as good reasons for use appear. Integrative medicine, according to this account, would be a concept depending on CAM, whereas traditional medicine would be fully independent.

Complementary and alternative would be independent of the label ‘traditional’ howsoever historically defined. Experimental treatment attempts would be separated from CAM based on their initial evidence-based plausibility, i.e. by not fulfilling the inclusion criterion (B).

One conceivably tempting appeal of the still remaining vagueness is that it is up to the respective agencies on national or international levels that execute guidelines on evidence assessment to add operational details, i.e. details about which kinds of evidence are sufficient to accept that a treatment idea is a good candidate for further testing. Admission boards could, for example, negotiate criteria for a model to be sufficiently analogous, for mechanisms to be sufficiently well-established and so on. Consensus between countries about these criteria would then generate areas where treatments have an unambiguous CAM status.

## Conclusion

According to evidence-based medicine, conventionality and acceptance should be based on the evidence available. We propose an accompanying sketch of a definition of CAM that centres around the notion of probable (in)effectiveness and thereby evidence deficiency. For a number of treatments, unconventionality and evidence deficiencies on various levels go hand in hand. This would be the case more often in a rational medical system and conventionality would ‘reply’ to well justified changes in the evidence standards. From this perspective, an evidence strategy definition of CAM, as sketched above, seems to be the more promising alternative to us when compared with a conventionality-based definition, a list of positive CAM attributes or the strategy to not define CAM at all. Our proposal has, however, to be improved and tested for consequences and acceptability given a suitable explanation of its advantages.

## Footnotes

1 This article elaborates and extends ideas published in Kremling (2024). We thank two anonymous reviewers for their critical comments and helpful suggestions, as well as Caspar Radunz for comments on an earlier version.

2 We will speak of ‘treatments’ throughout most of this article, though there are also techniques used for prevention or diagnosis that can be labelled as complementary or alternative.

3 Placebo effects, for example sugar pills in pain therapy, are, of course, non-pharmacological effects – but nevertheless real, though there was (Howick et al. 2013) and is an ongoing discussion about effect sizes, best description of mechanisms and clinical use (Ortega et al. 2022).

4 “There is a practical need to decide upon what can be considered [traditional and complementary medicine] for the purpose of treatment classification, health expenditure and so on. People creating databases must decide which treatments to store information about and how to categorise this information within subsets. Practical decisions need to be made about what is included in the education and training of health professionals that, to some degree, depends upon what falls within and what lies outside the domain of conventional medicine” (Chatfield and Kate 2018, 5).

5 The objections raised by Schöne-Seifert et al. against defining by conventionality or evidence will be taken into account later.

6 The authors’s starting point is a definition originally from the OAM (Office of Alternative Medicine), later NCCAM (National Center for Complementary and Integrative Health), now NCCIH (National Center for Complementary and Integrative Health), which is not officially used any more but is often quoted in older literature ( Zollman and Vickers (1999), [O’Connor et al. (1997)59], Carroll and Richard (2007); Chatfield and Kate (2018)): “Complementary and alternative medicine (CAM) is a broad domain of healing resources that encompasses all health systems, modalities, and practices and their accompanying theories and beliefs, other than those intrinsic to the politically dominant health system of a particular society or culture in a given historical period. CAM includes all such practices and ideas self-­defined by their users as preventing or treating illness or promoting health and well-being. Boundaries within CAM and between the CAM domain and that of the dominant system are not always sharp or fixed.” Today, the website only states: “If a non-mainstream practice is used together with conventional medicine, it’s considered “complementary. If a non-mainstream practice is used in place of conventional medicine, it’s considered “alternative.” (National Institute for Health 2023).

7 The authors of the article from 2022 have an idiosyncratic use of the expression ‘definition’: They call their list itself an “operational definition”. Instead, they should  as already mentioned above, describe the list as the ‘result’ of applying the unchanged 2011 definition.

8 PubMed alone lists 128 citations of the two mentioned publications (Wieland et al. (2011); Ng et al. (2022)) as of May 2025 – Google scholar lists even 479 citations.

9 “First we considered whether the historical notion of the therapy was CAM or conventional. As suggested above in the theoretical model of CAM, if the therapy was based upon the theories of a medical system outside the Western allopathic medical model, then (from the current perspective of the US and Europe, anyway) it would be labelled alternative medicine, or CAM.” (Wieland et al. 2011, 5).

10 See also NIH National Library of Medicine (2024): “Therapeutic practices which are not currently considered an integral part of conventional allopathic medical practice.”

11 “CAM procedures can primarily be defined via their distance to science-oriented medicine” (Anlauf et al. 2015, 2). It is not exactly clear whether this distance is part of the definition the authors suggest or a criterion that they estimate to have the best overlap with treatments that are factually called CAM. We suspect the latter. (Smith et al. 2016) seems to favour an evidence strategy but provides no “definitional nuances” (60). Teichfischer and Münstedt (2011) mostly describe possible differences but conclude (p. 20) that the basis of experience on which a treatment is based might be a good candidate – without further reflection on what this means in terms of definitions.

12 The EMB+ movement has argued for acknowledging knowledge about mechanisms systematically when evaluating claims of effectiveness (see Wilde (2023); Anjum et al. (2018)), claiming that is an expansion of the concept of (medical) evidence.

13 The considerations above had the ‘lack’ of evidence as the starting point for defining CAM. There is another option though, which has for example some proponents in Germany, especially oncology. Alternative medicine in this field seems to be implicitly defined by evidence of non-efficacy, whereas “complementary medicine” is defined as treatments with proven effectiveness as treatment additional to first-line therapies, for example physical training to reduce cancer induced fatigue (Leitlinienprogramm Onkologie 2023). This idea has not yet reached the level of an explicit definition and, consequently, can not be assessed here. We just want to point at a possible weakness: Boundary cases have not been discussed systematically. Is not every care measure (e.g. each change of bed-sheets) complementary treatment according to this proposal? The extension of the term ‘complementary’ might become way to large. Symptomatically, the newest respective guideline does not contain any comments about in- and exclusion criteria for the interventions assessed. This leaves the impression of an unsystematic sampling of the treatments evaluated.

14 The following list is a partially interpreted and reorganized translation, cf. (Schöne-Seifert et al. 2015, 240-241:).
